# Unusual cause of progressing dysphonia and stridor in a child

**DOI:** 10.1016/j.bjorl.2023.101308

**Published:** 2023-08-19

**Authors:** Marina Paese Pasqualini, Mariele Bressan, João Pedro Neves Lubianca, Talita Lopes Silva, Germana Viana Gomes Foinquinos, José Faibes Lubianca Neto

**Affiliations:** a Universidade Federal de Ciências da Saúde de Porto Alegre, Hospital da Criança Santo Antônio, Serviço de Otorrinolaringologia Pediátrica, Porto Alegre, RS, Brazil; b Universidade Federal do Rio Grande do Sul, Porto Alegre, RS, Brazil; c Hospital Vida e Saúde, Santa Rosa, RS, Brazil; d Laboratório Medicina Digital, Departamento de Imuno-histoquímica, Porto Alegre, RS, Brazil; e Universidade Federal de Ciências da Saúde de Porto Alegre, Departamento de Clínica Cirúrgica e Programa de Pós-Graduação em Pediatria, Porto Alegre, RS, Brazil

## Introduction

Laryngeal schwannomas are encapsulated tumors composed entirely of benign neoplastic Schwann cells,^1^ arising from the internal branch of the superior laryngeal nerve after it penetrates the thyrohyoid membrane.^2^ Schwannoma located within the larynx is uncommon, accounting for 0.1% of all benign neoplasms of this region.^2^

Laryngeal schwannomas usually present as insidious, slow-growing, submucosal masses and are most commonly seen between the ages of 20 and 50.^1^ This tumor is very uncommon in the pediatric population, with only 6 reported cases to date.^2^ The most common presentation was dysphonia, followed by dysphagia, dyspnea and foreign body sensation.^1^

Most schwannomas were located in the false vocal cords or in the aryepiglottic folds.^1^ and their differential diagnosis include laryngeal cyst, laryngoceles, chondromas, adenomas, mucoceles, lipomas or neuroﬁbromas.^2^

The treatment of choice is surgical excision with clear margins and the final diagnosis can only be confirmed through histopathology. The overall prognosis and outcome for laryngeal schwannoma is good.^2^

We intend to call attention for a very rare case of dysphonia and stridor in a 7-year-old girl that should be included in the differential diagnosis.

## Case report

**Figure 1:**
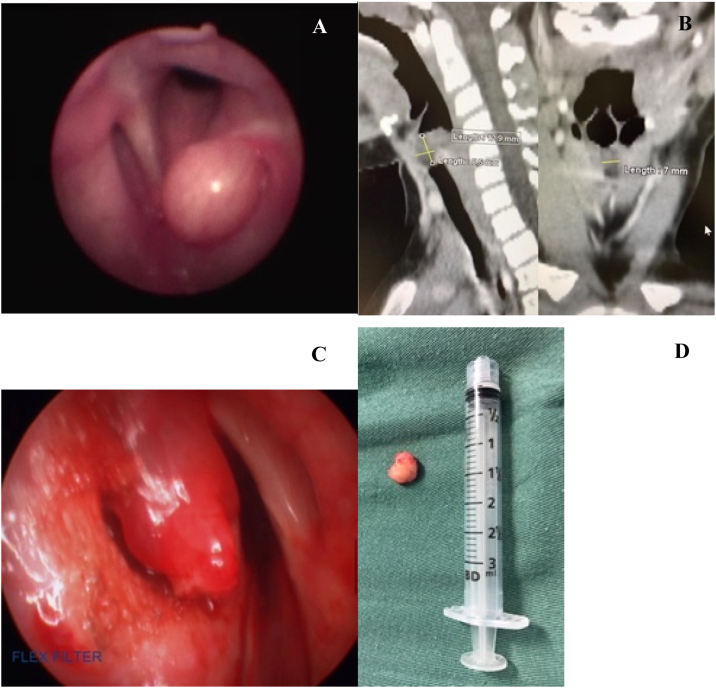
(A) Flexible fiber-optic laryngoscopy revealing submucosal swelling in the left ventricle. (B) Computed Tomographic (CT) scan of the neck revealing a submucosal hypodense supraglottic mass on the left side of the airway, measuring 8.5 × 12.9 × 7 mm. (C) View of the rigid laryngoscopy after removal of the submucosal mass with the preservation of the mucosal flap and (D) macroscopic appearance of the excised lesion.

**Figure 2:**
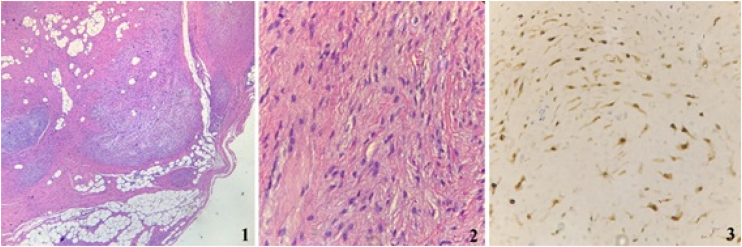
Histopathologic examination showing (1) biphasic pattern (H&E, 100×); (2) cells features (H&E, 400×) and (3) S100 immunoreactivity (400×).

A 7-year-old girl presented with one-year progressing dysphonia and a recent (1-month) appearing mild stridor. She had no significant medical history. Flexible fiber-optic laryngoscopy revealed a submucosal lesion in the left ventricle region (Fig. 1A). The vocal folds were free of lesions and had normal motion. There were no other significant findings in the head and neck examination. A Computed Tomographic (CT) scan of the neck revealed hypodense cystic mass in the left ventricle with extension to the vocal fold, with no enhance by the contrast, without significative mass effect and measuring 8,5 × 12,9 × 7 mm (Fig. 1B). The patient underwent resection micro laryngoscopy, and a submucosal solid, not cystic, mass was observed in the left laryngeal ventricle. The mass was easily dissected submucosally (Fig. 1C) and excised completely (Fig. 1D), then sent for a histopathologic examination (Fig. 2). The postoperative course was uneventful, and the last flexible fiber-optic laryngoscopy 2-years after the resection showed no detectable laryngeal lesion and preservation of the vocal fold mobility, with normal voice.

## Discussion

In 1987, Stanley et al. described two pediatric cases – 12-year-old female with hoarseness and 17-year-old male with other voice abnormality.^3^ In the same year, Phang et al. reported a 4-year-old-boy presented with inspiratory and expiratory stridor, which was getting progressively worse, when an emergency tracheostomy was done. Direct laryngoscopy showed a mass occupying the right side of the supraglottis.^4^ According to a recent systematic review and a case report, there are other 3 cases related to the date.^1^, ^5^

The most common presentation is related to vocalization, with hoarseness or dysphonia experienced by most patients (71.2%), followed by dysphagia (24.7%), dyspnea (23.3%), and foreign body sensation (16.4%). The presented case had one-year progressing dysphonia, seeking medical consultation only when presented mild stridor.^1^

Most schwannomas are located in the false vocal cords (45.8%) or the aryepiglottic folds (33.3%) with less frequent involvement of the true vocal folds (16.7%), epiglottis (9.7%), subglottic areas (5.6%), and post cricoid areas (4.1%).^1^ In the related case, the tumor presented in the left false vocal cord. Differential diagnoses that need to be considered include laryngeal cyst, laryngoceles, chondromas, adenomas, mucoceles, lipomas and neuroﬁbromas.^2^

The imaging of schwannomas shows typical features of a benign lesion: oval shape, regular margins, and absence of osseocartilaginous erosion and compression without infiltration of surrounding structures,^1^ like in the reported case. Schwannoma often exhibit heterogenic density on contrast enhancement, with centrally distributed areas of low attenuation, surrounded by a peripheral enhancing ring, finding that may have confused the radiologist, who interpreted the lesion as cystic. CT or MRI are not diagnostic and cannot always differentiate schwannomas from other benign tumors of the larynx.^2^

The diagnosis of certainty is given by histopathology, based on the Enger and Weiss histological schwannoma diagnosis criteria: (1) Encapsulated tumor; (2) The presence of Antoni A and/or Antoni B stroma; and (3) A positive S100 immunostaining.

Indications for surgical treatment are tumor growth and presentation of symptoms.^1^ Progressive dysphonia and recent onset stridor were the surgical indications in the case. Excision through micro laryngoscopy is recommended for small lesions as it confers the advantage of reducing the risk of injury to the Superior Laryngeal Nerve (SLN) and the Recurrent Laryngeal Nerve (RLN).^2^

That was the chosen approach, with elevation of a micro flap and submucous dissection of the mass. Other alternatives of external excision are laryngofissure, lateral pharyngotomy or lateral thyrotomy.^5^

The prognosis for laryngeal schwannoma is extremely good.^2^ Given the early presentation of residual disease, the latest literature review suggests performing a fibro laryngoscopy every 3-months for the first year and then annually for at least 2-years after surgical intervention.^1^

Our last revision fibro laryngoscopy was done with 2-years of follow-up and there is no sign of recurrence.

## Conclusion

Schwannoma within the larynx is rare and can present with a variety of symptoms ‒ from mild dysphonia to life threatening respiratory distress. We present a case with these both extremes’ symptoms: one-year progressing dysphonia and a one month appearing mild stridor. Diagnosis can only be conﬁrmed through histopathology. Surgical excision with clear margins remained as the treatment of choice, and should be done endoscopically, if possible, like in the presented case. The overall prognosis and outcome for laryngeal schwannoma is good, as demonstrated by the 2-year follow-up with no symptoms and no signs of recurrence.

## Ethical standards

We state that there was no submission of the case report ‒ Unusual cause of progressing dysphonia and stridor in a child ‒ to the Ethics Committee, since there is no demand from the main institution. Both patient and the responsible relative agreed with the discussion and publication of the case.

## Funding

This paper has no funding source.

## Conflicts of interest

We state that there are no known conflicts of interest associated with this publication and that there was no significant financial support for this work that could have influenced its outcome.

We authorize the publication of the article and declare that it is unpublished and has not been submitted in another journal.

We confirm that the manuscript has been read and approved by all the named authors and that there are no other people who meet the criteria for authorship who are not listed. We also confirm that the order of the authors listed in the manuscript has been approved by all.

We confirm that the appropriate intellectual property protection measures associated with this work have been provided and that there are no impediments to publication regarding intellectual property. In doing so, we confirm that we follow our institutions' regulations on intellectual property.

We further confirm that any aspect of the work covered in this manuscript that involved humans was conducted with the ethical approval of all relevant bodies and that these approvals are recognized in the manuscript.
